# Supplementary material for: Iso-acoustic focusing of cells for size-insensitive acousto-mechanical phenotyping
Source: Nat Commun. 2016 May 16;7:11556. doi: 10.1038/ncomms11556 (PMC4873643; doi:10.1038/ncomms11556)
Supplement: Supplementary Information — Supplementary Figures 1-11, Supplementary Notes 1-4 and Supplementary References [file ncomms11556-s1.pdf]

Supplementary Figure 1: Mapping the local acoustic field

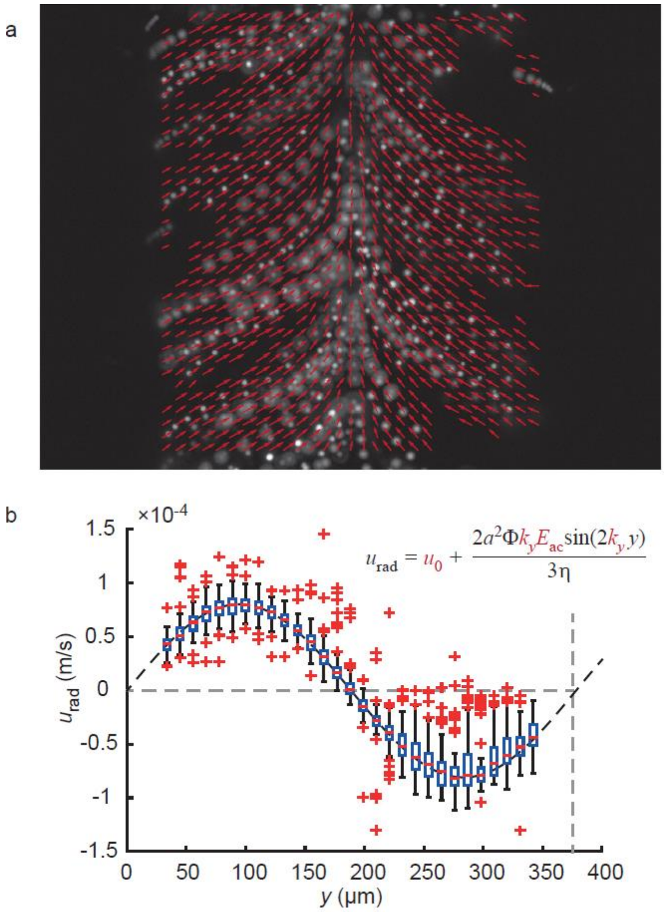

(**a**) Overlay of six consecutive frames of an image sequence of fluorescent microparticles (gray) submerged in 10 % iodixanol acquired immediately after turning on the acoustic actuation. Red arrows show velocity vectors from particle imaging velocimetry analysis of repeated onsets of sound. (**b**) Box plots of the *y*-components (u_{rad}), of the measured velocities vs position. For each box, the central mark (red) is the median, the edges of the box (blue) are the 25th and 75th percentiles, the whiskers extend to the most extreme data points not considered outliers, and outliers (red plus-sign) are defined as larger than $q_3 + 1.5(q_3 – q_1)$ or smaller than $q_1 – 1.5(q_3 – q_1)$, where q_1 and q_3 are the 25th and 75th percentiles, respectively. Data was fitted to a one-dimensional model $\underline{u}_{rad}$ of the acoustic field (black dashed line) with fitting parameters highlighted in red.

Supplementary Figure 2: **Acoustofluidic properties of iodixanol *versus* concentration**

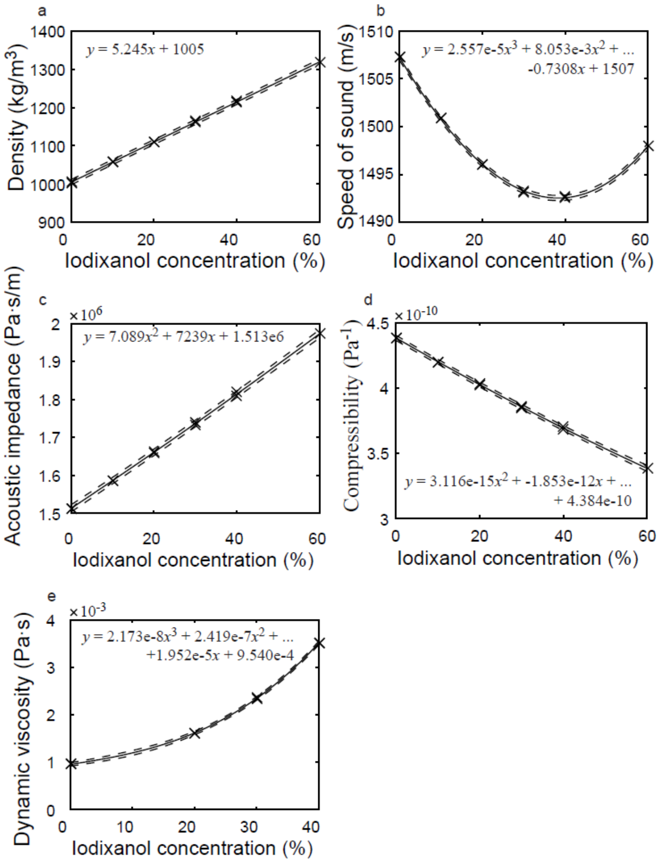

(**a**) Density ρ and (**b**) speed of sound c_s were measured simultaneously using a density and sound-velocity meter based on oscillating U-tube and ultrasound pulse echo respectively. (**c**) Compressibility ($\kappa = 1/\rho c_s^2$) and (**d**) acoustic impedance ($Z = \rho c_s = \sqrt{\rho/\kappa}$) were derived from the individual measurements of ρ and c_s. (**e**) Viscosity η was measured using a falling-ball viscometer. (**a-e**) Data was fitted to polynomials (solid lines). Dashed lines show the upper and lower 99% prediction bounds.

Supplementary Figure 3: Stop flow cell trajectories

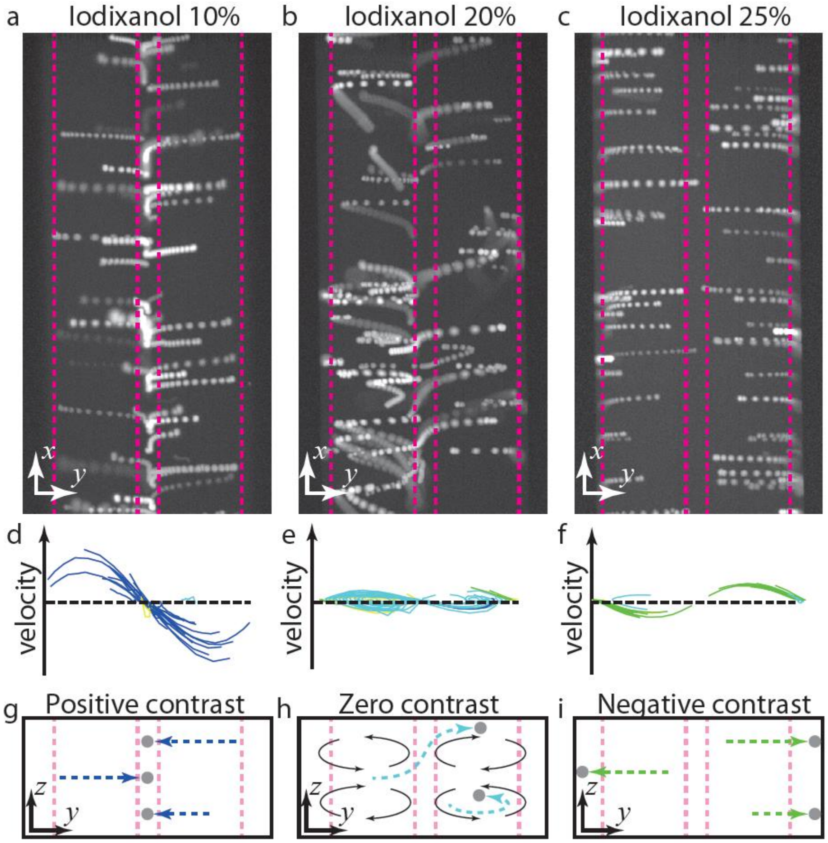

(**a-c**) Projection of cell images from 30 sequential frames for BA-F3 cells suspended in (**a**) 10 %, (**b**) 20 % and (**c**) 25 % iodixanol. Magenta dashed lines indicate the limits for the central region and the near-wall regions. (**d-f**) The corresponding cell velocities vs *y*-position corresponding to the tracks of cells in (**a-c**). Tracks are color coded based on the classification such that (**g**) blue indicate positive acoustic contrast, (**h**) cyan indicate zero acoustic contrast and (**i**) green indicate negative acoustic contrast. Yellow indicate unknown cells.

Supplementary Figure 4: Measured fluorescence intensity profiles for dextran dye.

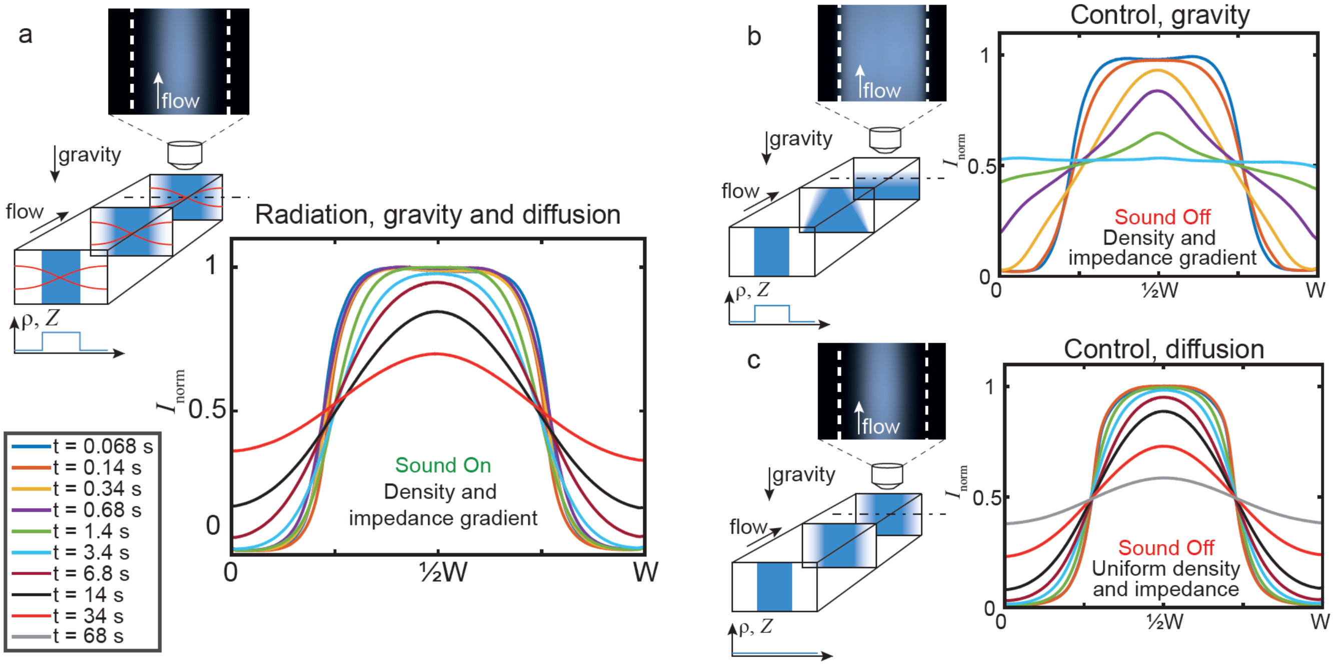

Fluorescence profiles imaged 20 mm downstream the trifurcation inlet for different retention times t obtained from Eq. (11) by varying the overall flow rate Q. (**a**) A density and acoustic impedance gradient is setup by an initial central and side inlet iodixanol concentration of 30% and 10%, respectively. The gradient is stabilized by the ultrasound, which counteracts both gravitational relocation and acoustic streaming in the bulk, such that the profile evolution is governed by diffusion. (**b**) Control experiment for gravitational relocation with the sound turned off. The profiles indicate that the central liquid reaches a stable position at the bottom of the channel within a few seconds. (**c**) Control experiment for diffusion only. The ultrasound is turned off and the iodixanol concentration was 20% in both center and side inlets so there is no density and acoustic impedance gradient present in the channel.

Supplementary Figure 5: Estimating the average acoustic energy density

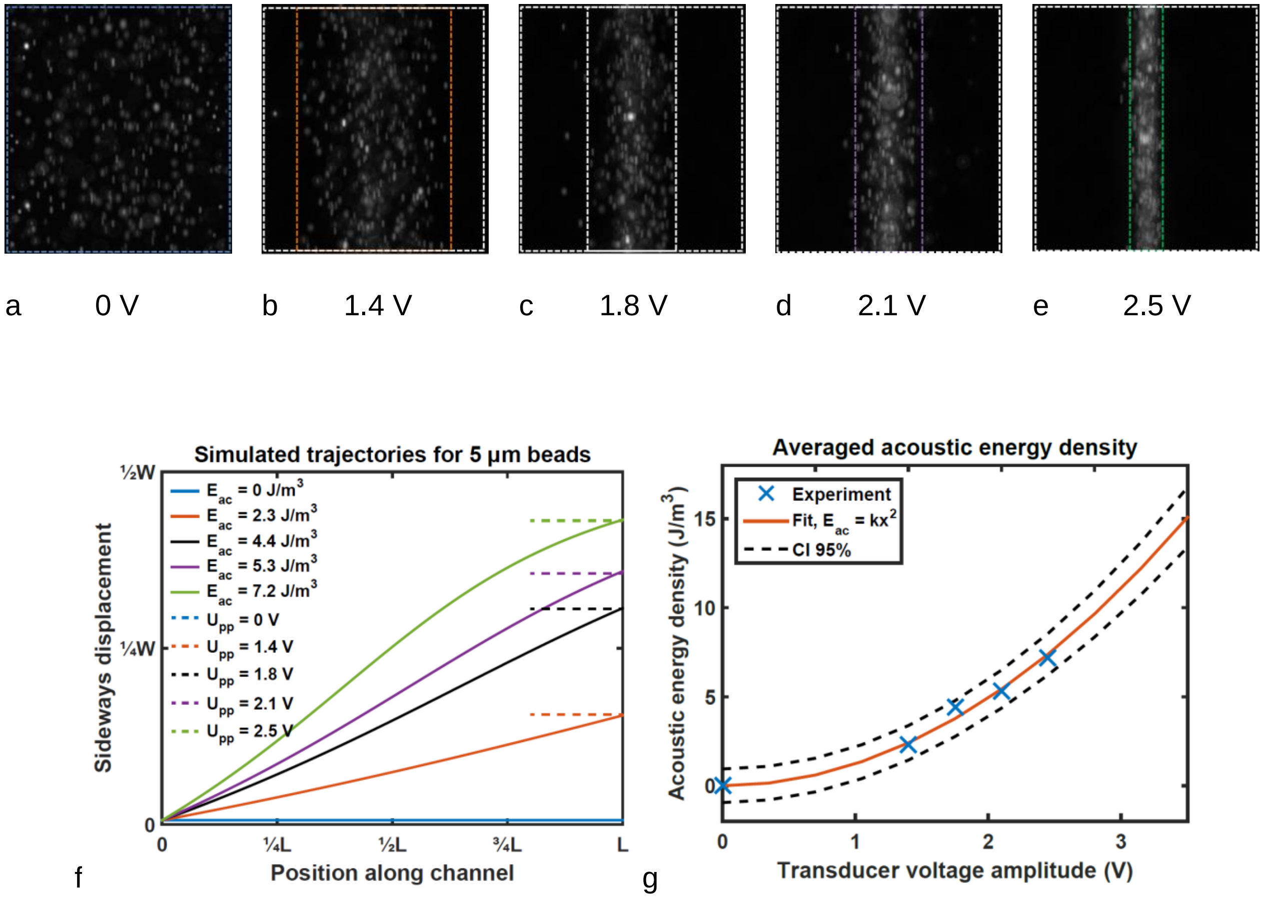

(**a-e**) Beads focusing in flow for increasing piezo actuator voltage amplitude. Regions (colored dashed lines) are chosen manually to encompass all beads except for some outliers. (**f**) Simulated trajectories of 5-µm-beads in a rectangular microchannel. Beads start near a wall and migrate towards $y = W/2$. Dashed lines show the position of the outermost observed beads from (**a-e**) at the end of the channel. Values for E_{ac} were adjusted iteratively in the simulation to match the observations. (**g**) Fit of E_{ac} versus U assuming a power 2 dependence.

Supplementary Figure 6: Time-dependent simulations of IAF cell trajectories in a diffusing iodixanol gradient

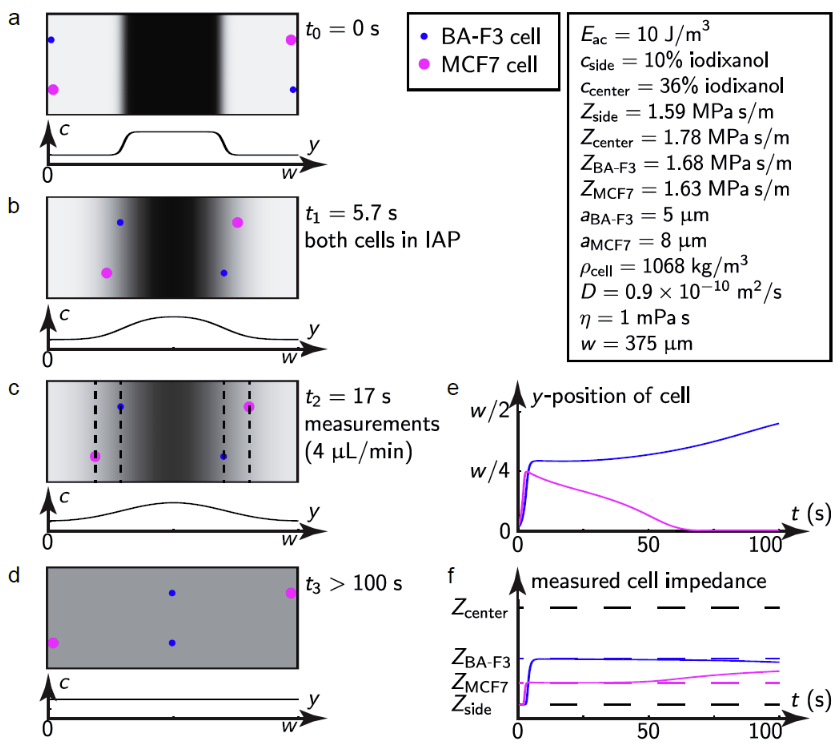

(**a**) Cells are introduced at time t_0 near the side walls at the inlet cross-section. (**b**) After initial migration caused by the acoustic force, the cells have reached their respective IAP at time t_1. (**c**) In the experiment, images are recorded at time t_2 with cells residing in their respective IAP. The cells stay in their diffusing IAP until diffusion has flattened the gradient. (**d**) At times longer than the diffusion time t_3, the gradient has vanished leaving the cells either at the center (postive contrast Φ) or at the walls (negative contrast Φ). (**e**) The transverse cell positions as function of time. (**f**) The measured cell impedances assuming $Z_{cell} = Z_{med}$ as a function of time. Accurate measurements with a relative error less than 0.3% are obtained at times t between 5.7 s and 51 s.

Supplementary Figure 7: Photo of the microfluidic chip and holder

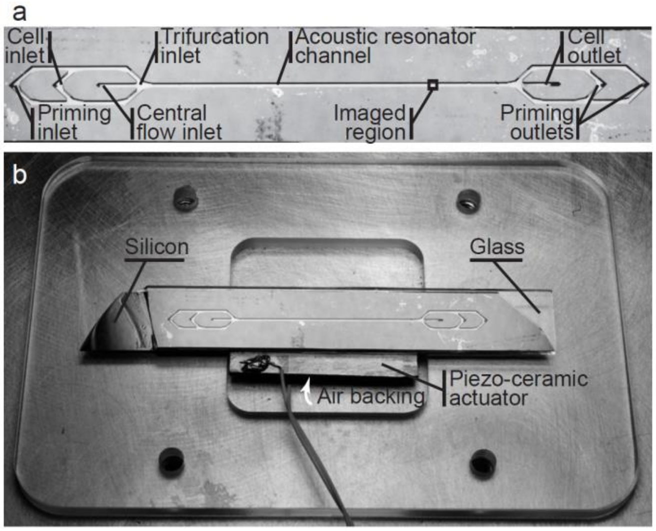

(**a**) Photo of the chip. (**b**) Photo of the chip in its holder with the piezoceramic actuator glued underneath.

Supplementary Figure 8: Flow cytometry of lymphocyte, granulocyte and monocyte preparations

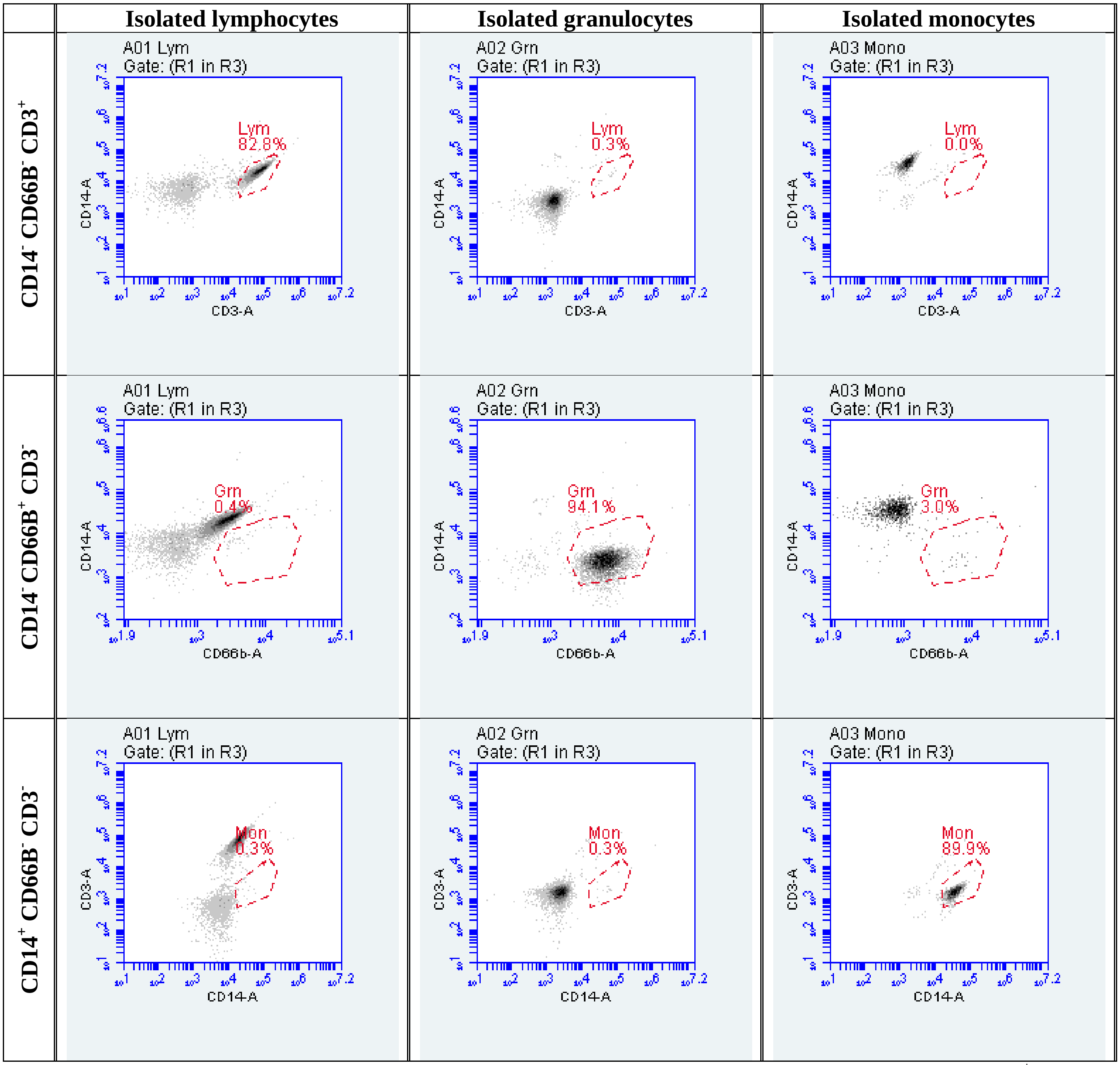

The purities of lymphocytes, granulocytes and monocytes are 82.8 %, 94.1%, and 89.9 % (gated on $CD45^{+}$), respectively. The purity of lymphocytes was underestimated given there were $CD3^{-}$ lymphocytes in the sample. According to the manufacturer manual (EasySep™ Direct Human Total Lymphocyte Isolation Kit, Stemcell Technologies), the percentage of CD3- lymphocyte can be around 9%.

Supplementary Figure 9: Standard curve for Dextran Cascade Blue fluorescence intensity in iodixanol

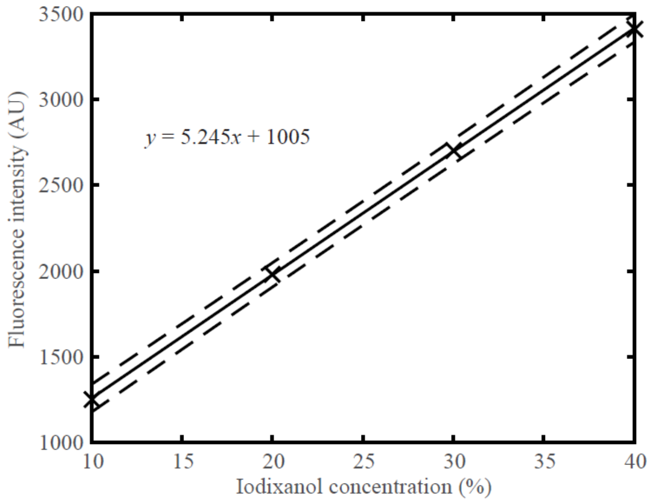

Fluorescence intensity of Dextran Cascade Blue as a function of iodixanol concentration. Solid line show linear fit and dashed lines show 99% prediction bounds.

Supplementary Figure 10: Relative impedance difference between the medium and the cell at the true IAP

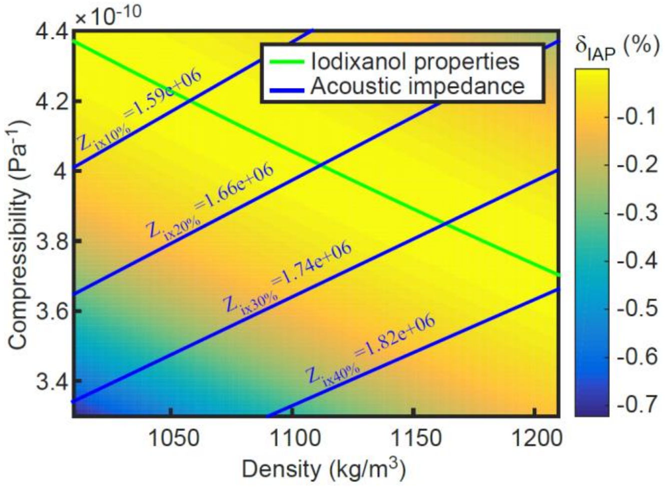

Color plot of the relative deviation $\delta_{\mathrm{IAP}} = (Z_{\mathrm{med}} - Z_{\mathrm{cell}})/Z_{\mathrm{cell}}$ at the true IAP ($\Phi = 0$) between the approximate cell impedance $Z_{\mathrm{cell}}^{\mathrm{approx}} \approx Z_{\mathrm{med}}$ and the actual value Z_{cell} as a function of cell density ρ_{c} compressibility κ_{c}. The green line represents the density and compressibility of iodixanol as a function of concentration. The four blue contour lines trace out the combinations of hypothetical cell properties (ρ_{c}, κ_{c}) that lead to the same acoustic impedance as iodixanol at concentrations of 10%, 20%, 30%, and 40%, respectively.

Supplementary Figure 11: Comparison of the temporal evolution of dextran dye concentration profiles in simulations and experiments.

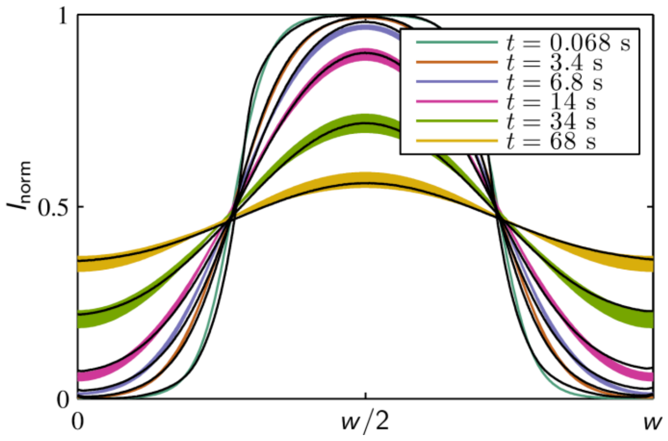

Concentration profiles at different times t. The colored bands show simulation results for the diffusivity D between 0.8×10^{-10} m^2 s^{-1} and 1×10^{-10} m^2 s^{-1} overlaid by black curves showing the normalized measured fluorescence intensity of dextran dye in 20% iodixanol (**Supplementary Fig. 4c**).

Supplementary Note 1: Basic theory of iso-acoustic focusing (IAF)

Consider the acoustic cavity in **Fig. 1a** of width w bounded by acoustically hard walls at $y = 0$ and $y = w$. By adjusting the sound frequency such that half the acoustic wavelength λ matches the width w, a standing wave builds up in the acoustically soft liquid. The resulting time-harmonic resonant pressure field p of amplitude p_a has an anti-node at each wall and a node at the channel center. Considering first the case of a homogeneous medium the field is given by $p = p_\mathrm{a}\cos(k_y y)$, where $k_y = 2\pi/\lambda$ is the wavenumber and $\lambda = 2w$. A small particle or cell of radius $a \ll \lambda$ suspended in this sound field will experience the acoustic radiation force $\boldsymbol{F}_\mathrm{rad}$ given by[1, 2],

$$\boldsymbol{F}_\mathrm{rad} = 4\pi a^3 \Phi k_y E_\mathrm{ac} \sin(2k_y y)\boldsymbol{e}_y\,, \quad (1)$$

where $E_\mathrm{ac} = \frac{1}{4}\kappa_\mathrm{m} p_\mathrm{a}^2$ is the time-averaged acoustic energy density and Φ is the acoustic contrast factor given by

$$\Phi = \frac{1-\tilde{\kappa}}{3} + \frac{\tilde{\rho}-1}{2\tilde{\rho}+1}. \qquad (2)$$

Φ depends on the ratios of cell-to-medium densities $\tilde{\rho} = \rho_\mathrm{c}/\rho_\mathrm{m}$ and adiabatic compressibilities $\tilde{\kappa} = \kappa_\mathrm{c}/\kappa_\mathrm{m}$, with the subscripts (c) and (m) denoting cell and medium, respectively. In general, Φ thus depends on four parameters.

In this work we deal with a medium stratified by the added iodixanol concentration c, which perturbs the density and compressibility across the y-direction. Hence, the contrast factor Φ depends on c through the concentration-dependent medium density $\rho_\mathrm{m}(c)$ and compressibility $\kappa_\mathrm{m}(c)$. These have been obtained from measurements of density and speed of sound as functions of iodixanol concentration, (**Supplementary Fig. 2**). The actual $\boldsymbol{F}_\mathrm{rad}$ is modeled by inserting $\Phi(c)$ in Eq. (1).

In IAF, cells move to the IAP of zero acoustic contrast defined by the iodixanol concentration c_IAP for which $\Phi(c_\mathrm{IAP}) = 0$. In general, the IAP of a cell thus depends on the two cell parameters ρ_c and κ_c. It would be valuable for the IAF method to allow identification of the IAP in terms of a single parameter. Here, we show that the acoustic impedance $Z = \sqrt{\rho/\kappa}$ may be used to quantify the IAP. We find, that in the IAP the effective acoustic impedance of the cell Z_cell equals that of the surrounding medium Z_med to within 0.8% for all combinations of cells with ρ_c ranging from 1000 kg m^{-3} to 1200 kg m^{-3} and κ_c ranging from 3.4 ×10^{-10} Pa^{-1} to 4.4 ×10^{-10} Pa^{-1}. To arrive at this conclusion, we input the cell parameters ρ_c and κ_c into the expression for the contrast factor in Eq. (2), then, using the fitted polynomials for $\rho_\mathrm{m}(c)$ and $\kappa_\mathrm{m}(c)$, we solve for the iodixanol concentration c_IAP satisfying $\Phi(c_\mathrm{IAP}) = 0$, which defines the true IAP. In **Supplementary Fig. 10** we then plot the relative impedance difference ($\delta_\mathrm{IAP} = (Z_\mathrm{med} - Z_\mathrm{cell})/Z_\mathrm{cell}$) between the medium and the cell at the true IAP as functions of cell density and compressibility. As can be anticipated, the error is zero for cells that have combinations of ρ_c and κ_c that exactly match the properties of the medium (the green contour line). For all other combinations in the experimentally relevant range, the error stays below 0.8%, demonstrating that the IAP is well-described by the simple impedance condition $Z_\mathrm{cell} = Z_\mathrm{med}$.

We can obtain the result more formally by writing Φ in terms of the relative compressibility and impedance. Extending the fraction in the second term of Eq. (2) by $1/\tilde{\kappa}$, we find in terms of $\tilde{Z} = Z_\mathrm{cell}/Z_\mathrm{med}$ that

$$\Phi = \frac{1-\tilde{\kappa}}{3} + \frac{\tilde{Z}^2 - 1/\tilde{\kappa}}{2\tilde{Z}^2 + 1/\tilde{\kappa}} \quad . \qquad (3)$$

In the true IAP where $\Phi = 0$, we may then solve for $\tilde{Z}$,

$$\tilde{Z} = \sqrt{\frac{2+\tilde{\kappa}}{(5-2\tilde{\kappa})\tilde{\kappa}}}\,, \quad \text{for } \Phi = 0\,. \qquad (4)$$

For the relative compressibility we further write $\tilde{\kappa} = 1 + \Delta\tilde{\kappa}$ and Taylor expand to lowest order in $\Delta\tilde{\kappa} \ll 1$. One finds

$$\tilde{Z} = 1 + \frac{1}{3}(\Delta\tilde{\kappa})^2\,, \quad \text{for } \Phi = 0\,, \qquad (5)$$

which shows that the leading error in assuming $Z_{\mathrm{cell}} = Z_{\mathrm{med}}$ (or $\tilde{Z} = 1$) is $\frac{1}{3}(\Delta\tilde{\kappa})^2$ and of second order in the relative compressibility difference $\Delta\tilde{\kappa}$. A relative difference of 30% thus gives an error of 3%. However, even with the full parameter range of **Supplementary Fig. 10**, the relative compressibility difference in the IAP never exceeds 15%, and thus the error is less than 0.8% for the full range of experimental conditions considered.

Supplementary Note 2: A scaling analysis of the acoustic stabilization of inhomogeneous liquids

In a homogeneous fluid the time-averaged acoustic energy density E_{ac} of the standing wave is constant across the channel width, and as a result there are no time-averaged net forces acting on the fluid due to the acoustic field. This is no longer the case in an inhomogeneous fluid, where gradients in density and compressibility lead to spatial inhomogeneities in the acoustic energy density. Since the relative change in the speed of sound of iodixanol concentrations is small in comparison to the relative change in the density (**Supplementary Fig. 2a-b**), we consider a fluid of density $\rho_{\mathrm{m}}(y) = [1 + \delta(y)]\rho_{\mathrm{m0}}$, where $\delta(y)$ is the position-dependent relative density variation (of the order 0.1) and ρ_{m0} is a constant reference density. The order-of-magnitude of the acoustic body force f_{ac} acting on the fluid can then be estimated as,

$$|f_{\mathrm{ac}}| \approx \partial_y E_{\mathrm{ac}} \approx \frac{\partial E_{\mathrm{ac}}}{\partial \rho_{\mathrm{m}}} \partial_y \rho_{\mathrm{m}} \approx E_{\mathrm{ac}}\, \partial_y \delta \,. \qquad (6)$$

In the channel of height h with horizontal walls at $z = 0$ and $z = h$, the hydrostatic pressure in the fluid is given by $p_{\mathrm{hyd}} = g(h - z)\rho_{\mathrm{m}}(y)$, where $g = 9.8\ \mathrm{m\ s^{-2}}$ is the acceleration due to gravity. Consequently, the force on the fluid along the y-direction due to hydrostatic pressure is

$$f_{\mathrm{hyd}} = -\,\partial_y p_{\mathrm{hyd}} = -g(h - z)\rho_{\mathrm{m0}}\, \partial_y \delta \,, \qquad (7)$$

with the maximum force at the bottom of the channel in $z = 0$. We obtain the estimated condition for the fluid to be stable against gravity from the requirement that the magnitude of the acoustic force $|f_{\mathrm{ac}}|$ is everywhere greater than $|f_{\mathrm{hyd}}|$. Since both f_{ac} and f_{hyd} are proportional to the gradient $\partial_y \delta$ the condition simply becomes

$$E_{\mathrm{ac}} > \rho_{\mathrm{m0}} g h \approx 2\ \mathrm{J\ m^{-3}} \,. \qquad (8)$$

The numerical value is calculated for our typical experimental conditions, $\rho_{\mathrm{m0}} = 1.1 \times 10^3\ \mathrm{kg\ m^{-3}}$ and $h = 150\ \mu\mathrm{m}$, and it is in agreement with our experimental measurements.

The experiments show that boundary-driven acoustic streaming in the bulk (Rayleigh streaming) is suppressed in an inhomogeneous fluid. This can be understood by a scaling argument comparing f_{ac} to the magnitude f_{str} of the shear-force density associated with the streaming flow rolls driven by the usual slip-velocity $v_{\mathrm{str}} = \Psi \frac{v_{\mathrm{a}}^2}{c_s}$ at the walls. Here, v_{a} is the amplitude of the acoustic velocity field, c_s is the speed of sound, and Ψ is a geometrical prefactor, which is equal to 3/8 for a planar wall[3]. A scaling estimate for the shear-force density is then,

$$|f_{\mathrm{str}}| \approx \eta \nabla^2 v_{\mathrm{str}} \approx \eta \frac{1}{L^2} \Psi \frac{v_{\mathrm{a}}^2}{c_s} \approx \frac{4\Psi\eta}{\rho_{\mathrm{m0}} c_s L^2} E_{\mathrm{ac}} \,, \qquad (9)$$

where L is the characteristic length scale, and it has been used that $E_{\mathrm{ac}} \approx \frac{1}{4}\rho_{\mathrm{m0}} v_{\mathrm{a}}^2$. The ratio of the destabilizing streaming force $|f_{\mathrm{str}}|$ in Eq. (9) and the stabilizing acoustic force $|f_{\mathrm{ac}}|$ in Eq. (8) using $L = h/4$ becomes

$$\frac{|f_{\mathrm{str}}|}{|f_{\mathrm{ac}}|} \approx \frac{|f_{\mathrm{str}}|}{E_{\mathrm{ac}}\delta/L} \approx \frac{4\Psi\eta}{\rho_{\mathrm{m0}} c_s L \delta} \approx \frac{16\Psi\eta}{\rho_{\mathrm{m0}} c_s h \delta} \approx 10^{-4} \,. \qquad (10)$$

Thus the scaling calculation shows that acoustic streaming is suppressed in the bulk of inhomogeneous fluids due to the density-gradient-induced acoustic force f_{ac}.

Supplementary Note 3: Numerical model of cell trajectories in diffusing iodixanol gradients

To confirm that our measurements at flow rates 4 µL min^{-1} and 8 µL min^{-1} do indeed yield the correct acoustic impedances Z_{cell} of the cells, we solve a simple numerical model of cell trajectories in the diffusing iodixanol gradient. An important question we address, is whether the acoustic force on the cell, which approaches zero near the IAP, is able to keep the cell in its IAP at intermediate time scales where the IAP moves due to diffusion.

Because the molecular diffusivity of the fluorescent dextran tracer and the iodixanol are nearly identical, we assume, as discussed in the main text (**Main article, Creating an acoustic impedance gradient**), that the measured fluorescence signal is proportional to the iodixanol concentration as it evolves in time. The experimentally measured diffusive evolution of the fluorescence profile in 20% iodixanol (**Supplementary Fig. 4c**) is well described by Fickean diffusion of the concentration c,

$$\partial_t c = D\nabla^2 c, \quad (11)$$

with zero flux at the channel walls. Treating the diffusivity D as a free parameter in our numerical model, we obtain the best agreement with experiments using the value $D = 0.9 \times 10^{-10}$ m^2 s^{-1} (**Supplementary Fig. 11**).

Considering the cell trajectories, we solve for the transverse dynamics along the y-direction neglecting effects of inertia (Reynolds number $\sim 10^{-3}$), gravitational sedimentation and acoustic streaming. The dynamical equation for the cell then takes the form $F_{\mathrm{rad}} + F_{\mathrm{drag}} = 0$, with F_{rad} given by Eq. (1) and the Stokes drag given by $F_{\mathrm{drag}} = -6\pi\eta a\, \partial_t y$, where η is the dynamic viscosity of the medium. This leads to the following equation for the cell position y

$$\partial_t y = \frac{2k_y a^2 E_{\mathrm{ac}}}{3\eta} \Phi(c(y)) \sin(2k_y y). \qquad (12)$$

Note that the contrast factor $\Phi = \Phi(c(y))$ is a function of y through the diffusing iodixanol concentration c. It is calculated from Eq. (2) using the measured polynomials for $\kappa_{\mathrm{m}}(c)$ and $\rho_{\mathrm{m}}(c)$, **Supplementary Fig. 2**. The time-dependent solution of Eqs. (11) and (12) was carried out in COMSOL Multiphysics using the Particle Tracing Module to integrate the cell trajectory.

Using this model, we simulated cell trajectories of BA-F3 and MCF7 cells starting at time $t = 0$ a radius a from the sidewall in $y = 0$. The side and central streams were of initial iodixanol concentration 10% and 36%, respectively. The cell densities were set to $\rho_{\mathrm{c}} = 1068$ kg m^{-3} as previously reported for MCF7 cells[4], while the compressibilities κ_{c} were calculated from the measured cell impedances Z_{c}.

The simulation results (**Supplementary Fig. 6**) summarize the IAF method: In IAF, cells are introduced near the sides of the channel walls in a low acoustic-impedance medium (**Supplementary Fig. 6a**). When subject to the acoustic field in the cavity, the cells migrate rapidly to reach their IAP in the initially steep impedance gradient at the border of the central flow stream (**Supplementary Fig. 6b**). The gradient decreases slowly over time due to molecular diffusion of the iodixanol. During this process, the acoustic force maintains the cells at their respective IAP's allowing accurate measurements of the cell impedances (**Supplementary Fig. 6c**). Finally, when diffusion has eliminated all gradients, the cells are located either at a channel wall or at the channel center depending on their acoustic impedance relative to the acoustic impedance of the homogenized medium (**Supplementary Fig. 6d**).

The transverse cell position as a function of time is plotted in **Supplementary Fig. 6e** and shows the rapid migration in the first few seconds followed by slower migration as the IAP changes due to diffusion. Combining the cell positions with the temporal development of the acoustic impedance profile of the medium gives the apparent IAP and cell impedance versus time plotted in **Supplementary Fig. 6f**. Clearly, there exists a temporal window $t_1 < t < t_3$ during which accurate measurements of the cell impedance Z_{cell} of both BA-F3 and MCF7

can be obtained. Tolerating a relative impedance error between the cell and the medium of maximum 0.3% we obtain the lower bound $t_{1,\mathrm{BA-F3}} = 5.7$ s and $t_{1,\mathrm{MCF7}} = 3.0$ s. For the upper bound we find $t_{3,\mathrm{BA-F3}} = 79$ s and $t_{3,\mathrm{MCF7}} = 51$ s. Consequently, accurate measurement of the acoustic impedance of both BA-F3 and MCF7 cells can be obtained for $5.7\ \mathrm{s} < t < 51$ s corresponding to flow rates between 1.3 µL min^{-1} and 12 µL min^{-1}.

In conclusion, the simulations have shown that the IAF method represents a robust method of obtaining the acoustic impedance of the cells in our experiments. The experimental flow rates of 4 µL min^{-1} and 8 µL min^{-1} ensure that the cells are indeed located in their IAP at the time of measurement.

Supplementary Note 4: Time-scale analysis of iso-acoustic focusing

As demonstrated by the simulation results in **Supplementary Fig. 6**, IAF involves three time scales. First, there is the time scale t_1 of the initial migration to the IAP due to the acoustic radiation force (**Supplementary Fig. 6b**). Second, there is the retention time t_2 of a cell in the channel set by the flow rate Q. Finally, there is the diffusion time t_3 over which the impedance gradient is flattened (**Supplementary Fig. 6d**). For the IAF method to work properly the time scales must be ordered $t_1 < t_2 < t_3$ such that cells are in their IAP when imaged near the outlet (**Supplementary Fig. 6c**). Below we give simple analytical estimates for these three time scales useful for deciding experimental design parameters such as channel dimensions and flow rates for various cell types.

The initial migration time scale t_1 can be estimated by integration of Eq. (12) from $t = 0$ to $t = t_1$ assuming a constant contrast factor Φ. Starting from an initial position $0 < y_0 < w/2$ and migrating to a final position y_1 at $y_0 < y_1 < w/2$ takes a time[1]

$$t_1 \approx \frac{3\eta}{4\Phi(k_y a)^2}\frac{1}{E_{\mathrm{ac}}}\ln\left[\frac{\tan[k_y y_1]}{\tan[k_y y_0]}\right] = 3.1\ \mathrm{s}, \qquad (13)$$

where the numeric value is calculated for an MCF7 cell with the parameters given in **Supplementary Fig. 6** assuming a side inlet stream of 10% iodixanol and using $y_0 = a$ and $y_1 = w/3$.

The characteristic diffusion time t_3 for diffusion over one third of the channel is

$$t_3 \approx \frac{(w/3)^2}{2D} = \frac{w^2}{18D} = 87\ \text{s}, \qquad (14)$$

again with the numeric value calculated using the parameters given in **Supplementary Fig. 6**.

The retention time t_2 is obtained from the flow rate as

$$t_2 = \frac{w \cdot h \cdot l}{Q} = 17\ \text{s}, \qquad (15)$$

where the numeric value is calculated for a flow rate of 4 μL min^{-1} and l = 20 mm is the downstream distance from the inlet to the imaging region. These simple estimates are in agreement with the simulation results of **Supplementary Fig. 6**.

Supplementary References

1. Barnkob R, Augustsson P, Laurell T, Bruus H. Measuring the local pressure amplitude in microchannel acoustophoresis. *Lab Chip* **10**, 563-570 (2010).

2. Yosioka K, Kawasima Y. Acoustic radiation pressure on a compressible sphere. *Acustica* **5**, 167-173 (1955).

3. Lord Rayleigh. On the circulation of air observed in Kundt's tubes, and on some allied acoustical problems. *Philosophical Transactions of the Royal Society of London* **175**, 1-21 (1884).

4. Hartono D, Liu Y, Tan PL, Then XYS, Yung L-YL, Lim K-M. On-chip measurements of cell compressibility via acoustic radiation. *Lab Chip*, (2011).

5. Barnkob R, Augustsson P, Laurell T, Bruus H. Acoustic radiation- and streaming-induced microparticle velocities determined by microparticle image velocimetry in an ultrasound symmetry plane. *Phys Rev E* **86**, (2012).

6. Augustsson P, Barnkob R, Wereley ST, Bruus H, Laurell T. Automated and temperature-controlled micro-PIV measurements enabling long-term-stable microchannel acoustophoresis characterization. *Lab Chip* **11**, 4152-4164 (2011).
